# 

**Published:** 1913-10-25

**Authors:** 


### Appointments.

Mrs. Alice Stalker, M.B., Ch.B., D.P.H., has been appointed assistant medical officer and assistant tuberculosis officer at a salary of £275 by the Wigan Education Authority.

The Right Hon. Earl Derby, G.C.V.O., P.C., D.L., has consented to accept the office of President of the Twenty-ninth Congress of the Royal Sanitary Institute, to be held at Blackpool from July 6 to 11, 1914.

The Committee of Management of the Seamen's Hospital Society, acting upon the recommendation of the Medical Council, has appointed Colonel T. D. Collis Barry, I.M.S., physician in charge of the X-Ray Department of the *Dreadnought*. The X-Ray Department is about to be remodelled, many of the appliances contained therein re-adjusted, and new apparatus purchased.

The Rev. T. G. L. Davies, chaplain to the Worcester County and City Mental Hospital, has been appointed chaplain to the new Essex and Colchester Mental Hospital. As a cricketer, being captain of the institution cricket team, and as a member of the Asylum Workers' Association, Mr. Davies is a very active member of the staff. His successor in the office of chaplain is the Rev. F. D. Lane.

The Harley Institute.—If any member of the profession has received a card containing particulars of this Institute, with words relating to commission for applications made for nurses written thereon, we should be glad if they will communicate with the Editor of The Hospital, at the Office, 28 and 29 Southampton Street, Strand, London, W.C.

### BOOKS on HOSPITALS and INSTITUTIONS.

By SIR HENRY BURDETT, K.C.B., K.C.V.O.

### HOSPITALS & ASYLUMS OF THE WORLD.

In Four Volumes, with Portfolio of Plans. Complete, £12 12s.

Their Origin, History, Construction, Administration, Management and Legislation; with Plans of the chief Medical Institutions, accurately drawn to a uniform scale.

Vols. I. and II. (only).—Asylums and Asylum Construction, £6 15s.
Vols. III. and IV.—Hospitals and Hospital Construction—with Portfolio of Plans, £9.
The Portfolio of Plans (20in. by 14in.), separately, price £4 14s. 6d. Carriage extra.

IMPORTANT.—AS ONLY A FEW COMPLETE COPIES OF THE WORK REMAIN UNSOLD, Secretaries and Institution Librarians are recommended to take immediate steps to procure this Encyclopædia Britannica of Hospital and Institutional affairs.

### BURDETT'S HOSPITALS & CHARITIES.

The Year Book of Philanthropy, and Hospital Annual.

"The ideal of what a work of reference ought to be."—*The Times.*

Published Annually, over 1000 pages. 10s. 6d. net; 10s. 11d. post free.

This Annual is now in its Twenty-fourth Year, and the complete series forms a Library of Hospital Facts and Figures absolutely unobtainable from any other source. Everyone associated with or interested in our Hospitals and Charities should not only obtain the new edition, but all previous issues, since they are a valuable history of the Hospital movement throughout the English-speaking world.

### COTTAGE HOSPITALS, GENERAL, FEVER AND CONVALESCENT.

Their Progress, Management and Work in Great Britain, Ireland and the United States.

Third Edition, profusely illustrated, with nearly 50 Plans, Diagrams, etc.
Cloth gilt. 10s. 6d. net.

A New and Abridged Edition of this book is now in preparation and will be published shortly.

### THE UNIFORM SYSTEM OF ACCOUNTS.

For Hospitals and all Classes of Public Institutions.

With Special Forms of Accounts and Specimen Rulings of a complete set of books.
New Edition. In the Press.

### ACCOUNT BOOKS FOR INSTITUTIONS.

Designed in accordance with the Uniform System.

(Full description of Books and Prices on application.)

### MEDICAL ATTENDANCE OF LONDONERS.

An Economical System of Medical Relief freed from existing abuses and adequate to protect all classes.

In paper cover. 1s. post free.

### HOW TO BECOME A NURSE.

The Nursing Profession. How and Where to Train.

A Complete Guide to Training for the calling of a Nurse, with particulars of Nurse Training Schools in the United Kingdom and Abroad.

Price 2s. net. By Post, 2s. 4d.

"The advice to women desirous of becoming Nurses is simple sound and discreet."—*Lancet.*

### HOW TO SUCCEED AS A TRAINED NURSE.

Being a Guide to Remunerative Employment, with particulars of the various openings in the United Kingdom and Abroad, Government and Municipal departments, Associations and Co-operations where the services of private Nurses are in demand.

Price 2s. 6d. By Post, 2s. 9d.

Of all Booksellers, or of
**The Scientific Press, Limited,** 28/29 SOUTHAMPTON STREET, STRAND. LONDON, W.C.

